# RPS27a and RPL40, Which Are Produced as Ubiquitin Fusion Proteins, Are Not Essential for p53 Signalling

**DOI:** 10.3390/biom13060898

**Published:** 2023-05-28

**Authors:** Matthew John Eastham, Andria Pelava, Graeme Raymond Wells, Nicholas James Watkins, Claudia Schneider

**Affiliations:** Biosciences Institute, The Medical School, Newcastle University, Newcastle upon Tyne NE2 4HH, UK

**Keywords:** ribosomal proteins, ribosome, ubiquitin, p53, ribosome biogenesis

## Abstract

Two of the four human ubiquitin-encoding genes express ubiquitin as an N-terminal fusion precursor polypeptide, with either ribosomal protein (RP) RPS27a or RPL40 at the C-terminus. RPS27a and RPL40 have been proposed to be important for the induction of the tumour suppressor p53 in response to defects in ribosome biogenesis, suggesting that they may play a role in the coordination of ribosome production, ubiquitin levels and p53 signalling. Here, we report that RPS27a is cleaved from the ubiquitin-RP precursor in a process that appears independent of ribosome biogenesis. In contrast to other RPs, the knockdown of either RPS27a or RPL40 did not stabilise the tumour suppressor p53 in U2OS cells. Knockdown of neither protein blocked p53 stabilisation following inhibition of ribosome biogenesis by actinomycin D, indicating that they are not needed for p53 signalling in these cells. However, the knockdown of both RPS27a and RPL40 in MCF7 and LNCaP cells robustly induced p53, consistent with observations made with the majority of other RPs. Importantly, RPS27a and RPL40 are needed for rRNA production in all cell lines tested. Our data suggest that the role of RPS27a and RPL40 in p53 signalling, but not their importance in ribosome biogenesis, differs between cell types.

## 1. Introduction

The production of ribosomes, the most energetically consuming process in the cell, is up-regulated in cancer, down-regulated in differentiated cells and blocked by many forms of cellular stress [1,2,3]. As expected for such an important pathway, ribosome production is controlled by and also controls the major signalling pathways in the cell, such as mTOR, c-Myc and p53 [4]. Even though ribosome production appears to be upregulated in cancer, ribosome biogenesis defects are also linked to multiple types of cancer [5,6,7]. Defects in ribosome biogenesis are further found in more than 20 genetic diseases, termed ribosomopathies, which include Diamond Blackfan anaemia and Treacher–Collins syndrome [8,9]. Interestingly, in several cases, some or all of the ribosomopathy symptoms have been shown to be p53-dependent [10,11]. Therefore, understanding how ribosome production is coupled to cellular signalling is key to understanding how defects in ribosome production cause disease.

Ribosomes consist of two ribosomal subunits (small (SSU, 40S) and large (LSU, 60S)) that contain four ribosomal (r)RNAs and about 80 ribosomal proteins. The 18S, 5.8S and 28S rRNAs are transcribed as part of a long precursor rRNA (pre-rRNA) by RNA polymerase I in the nucleolus. The 47S pre-rRNA undergoes extensive endo- and exonucleolytic processing to generate the mature rRNAs [12,13]. The fourth rRNA, the 5S rRNA, is transcribed by RNA polymerase III and joins the LSU as the 5S RNP, together with the ribosomal proteins RPL5 (also known as uL18) and RPL11 (uL5) [14]. The pre-rRNA is bound by a myriad of trans-acting factors and assembles with the ribosomal proteins to generate the functional ribosomal subunits [15,16]. Surprisingly, the majority of the ribosomal proteins are over-produced [17]. Ribosomal proteins are unstable outside the ribosome, and the excess proteins are turned over by the proteasome.

**Figure 1:**
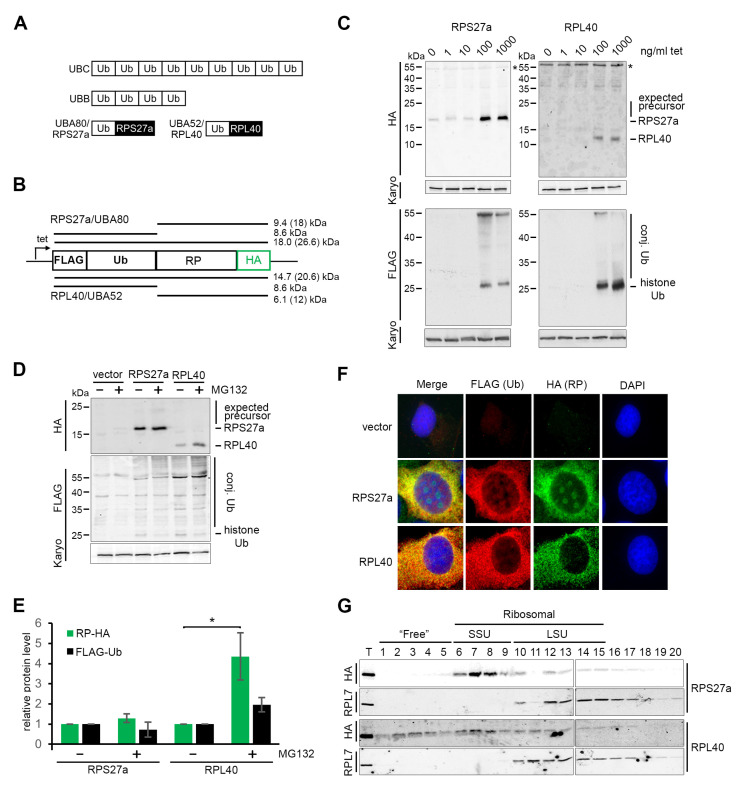
The stability and function of RPL40, but not RPS27a, are affected by the affinity tag. (**A**) Schematic representation of the organisation of the poly-ubiquitin (Ub) and ubiquitin–ribosomal protein fusion precursors. (**B**) Schematic representation of the ubiquitin (Ub)-ribosomal protein (RP) expression construct with N-terminal FLAG tag and C-terminal HA tag under the control of a tetracycline (tet)-regulated promoter. The bars and molecular masses above and below represent the expected fusion protein, and cleaved products, from the RPS27a and RPL40 cDNAs. Note that the HA-tagged ribosomal proteins appear slightly larger than expected (**C**), likely due to the basic nature of the amino acid sequence in each case and the gel-based mass for the HA-tagged ribosomal protein and expected mass for the fusion protein are shown in brackets. (**C**) U2OS cells stably expressing either RPS27a or RPL40 ubiquitin fusion precursor proteins under the control of a tetracycline-regulated promoter were incubated with 0–1000 ng/mL tetracycline (tet), as indicated above each lane. Proteins from these cells were harvested after 18 h and analysed by western blotting using antibodies that recognise the HA-tag, the FLAG-tag or karyopherin (Karyo; loading control), as indicated on the left of each panel. The positions of the various proteins and expected ubiquitin–ribosomal protein fusions are indicated on the right of the panels. * indicates a non-specific protein band detected by the anti-HA antibody. (**D**) U2OS cells stably expressing either the RPS27a or the RPL40 ubiquitin fusion precursor protein or the empty pcDNA5 vector were incubated for 18 h with 1000 ng/mL tetracycline in the absence (−) or presence (+) of 25 µM MG132. Proteins from these cells were analysed by Western blotting as described in panel (**C**). (**E**) The ribosomal protein-HA (RP-HA, green bars) and FLAG-Ub (black bars) Western blot signals from panel (**D**) were quantitated and normalised to karyopherin and plotted. Quantification is based on 3 independent repeats. Error bars indicate SEM. * < 0.05. (**F**) U2OS cells stably expressing either the RPS27a or the RPL40 ubiquitin fusion precursor protein or the empty pcDNA5 vector were analysed by immunofluorescence using antibodies that recognise the FLAG-tag (FLAG (Ub); red) or the HA-tag (HA (RP), green) or DAPI to visualise DNA (blue). (**G**) Whole-cell extracts from U2OS cells stably expressing either the RPS27a or the RPL40 ubiquitin fusion precursor protein were separated on 10–40% glycerol gradients. Fractions were analysed by western blotting, using an anti-HA antibody to detect the HA-tagged ribosomal protein and an anti-RPL7 antibody to visualise LSU complexes. Positions of free, non-RNP associated proteins (“Free”), 40S (SSU) and 60S (LSU) (pre-)ribosomal complexes are indicated.

Two eukaryotic ribosomal proteins, RPS27a (eS31; UBA80 gene) and RPL40 (eL40; UBA52 gene), are both encoded as C-terminal fusion proteins with ubiquitin. There are four ubiquitin-encoding genes in humans. Two are multi-copy ubiquitin repeats (UBB and UBC) and two are ubiquitin–ribosomal protein fusions (Figure 1A) [18]. The ubiquitin moiety is needed for Rps31 (yeast RPS27a) and Rpl40 production in yeast [19,20]. Ubiquitin, which makes up 0.5–1% of the total protein in the cell, is an 8.6 kDa polypeptide and a common post-translational modification, which attaches covalently to one or more lysine residues in the target protein [18]. There are multiple lysine residues in ubiquitin that can be used for linkage to proteins. Different ubiquitin linkage types have distinct functions, ranging from the modification of protein function to the degradation of the target protein [21]. The multicopy ubiquitin repeats and the ubiquitin–ribosomal protein fusions all need to be processed by yet-to-be-defined deubiquitinases to release the individual polypeptides. Interestingly, the deubiquitinase USP16 has recently been demonstrated to remove a regulatory mono-ubiquitin from an internal lysine within RPS27a. This likely represents a novel quality-control step in pre-40S maturation, but USP16 does not appear to process the RPS27a precursor fusion protein [22]. Importantly, the ubiquitin moiety would be expected to interfere with ribosome biogenesis and translation if it is not cleaved from the ribosomal protein [23]. Indeed, ubiquitin release has been shown to be required for the maturation and function of both the 40S and 60S subunits in yeast [19,24]. Interestingly, both genes encoding the ubiquitin–ribosomal protein fusions are preferentially over-expressed during hepatoma cell apoptosis [25].

Ribosome biogenesis directly controls p53 homeostasis [10,26,27,28,29]. The 5S RNP binds to and inhibits the E3 ubiquitin ligase MDM2. MDM2 suppresses p53 transcriptional activity and targets p53, through ubiquitination, for proteasomal degradation [30]. Defects in ribosome biogenesis result in 5S RNP accumulation and binding/inhibition of MDM2, leading to both p53 stabilisation and activation [26,27,28,29]. Multiple ribosomal proteins have been shown to bind MDM2, including RPL5, RPL11 and RPS27a, and to regulate its activity [10,31,32,33,34,35,36]. However, much of this work is based on protein over-expression, and only in a few cases has the endogenous protein been shown to bind to MDM2 [31,32,33,36]. RPS27a is one example where the endogenous protein has been shown to bind directly to MDM2 and suppress its activity [32]. Indeed, as seen with RPL5 and RPL11, knockdown of RPS27a inhibited actinomycin D (ActD; inhibits RNA pol I) and 5 fluorouracil (5FU; inhibits pre-rRNA processing) from activating p53 in U2OS cells [32]. However, the knockdown of most other ribosomal proteins causes a ribosome biogenesis defect that leads to p53 activation/stabilisation via the 5S RNP [28,29,37]. Tagged RPL40, expressed from a transfected plasmid, has also been shown to bind MDM2 and the data indicate that RPL40 regulates p53 together with the long non-coding (lnc)RNA LUCAT1 [38]. However, in several conflicting reports, the knockdown of either RPS27a or RPL40 was shown to induce p53 in HCT116 cells [29] and A549 cells [28,39]. This data suggests that RPS27a and RPL40 are not essential for p53 activation/stabilisation in response to defects in ribosome biogenesis in all cell types.

As ubiquitin and ribosome biogenesis are central to the regulation of the major cellular signalling pathways, especially p53 regulation, it is tempting to speculate that these fusion proteins couple the ubiquitin pathway to ribosome biogenesis and p53 signalling. It is further possible that the cleavage event separating ubiquitin from the ribosomal protein, as previously shown in yeast, may also be a key event in human ribosomal maturation.

Here, we have set up an in vivo system using stably transfected U2OS cells to investigate whether processing of the ubiquitin-ribosomal protein precursor protein is coupled to ribosome biogenesis. Comparing three different cell lines commonly used to study p53 signalling, we have also examined the roles of RPS27a and RPL40 in human ribosome biogenesis and cellular signalling to shed light on previous conflicting reports.

## 2. Materials and Methods

### 2.1. Cell Culture, RNAi and the Generation of Stable Cell Lines

U2OS cells were grown according to standard protocols at 37 °C with 5% CO_2_ in DMEM supplemented with 10% foetal calf serum and 1% penicillin/streptomycin. MCF7 cells were cultured using RPMI1640 media, 10% foetal calf serum and 1% penicillin/streptomycin. LNCaP cells were grown in RPMI1640 media with L-Glutamine, 25 mM HEPES, 10% foetal calf serum and 1% penicillin/streptomycin. U2OS Flp-In T-REx cell lines were a generous gift from Prof. Laurence Pelletier [40]. For the Flp-In system, the cDNAs for RPS27a and RPL40 were cloned into a pcDNA5 vector to enable expression of the proteins with an N-terminal 2xFLAG-PreScission protease site-His6 (FLAG) tag and a 2xHA tag at the C-terminus. These plasmids, or the empty pcDNA5 vector, were transfected into Flp-In T-REx U2OS cells and cells that had stably integrated the plasmid into their genome were selected using Hygromycin B, according to the manufacturer’s instructions (Invitrogen/Thermo Fisher). Expression of tagged proteins was induced by the addition of 0–1000 ng/mL tetracycline for 18–48 h prior to harvesting.

For RNAi-mediated knockdowns, the cells were transfected with siRNA duplexes (50 nM) using Lipofectamine RNAiMAX reagent according to the manufacturer’s instructions (Invitrogen/Thermo Fisher) as described previously [41]. For RPL40 and RPL7 knockdowns, Dharmacon smartpools were used. Individual siRNAs (Eurofins MWG) were used for RPL5 (5′-GGUUGGCCUGACAAAUUAUdTdT-3′ [42]), RPS19 (5′-GAUGGCGGCCGCAAACUGUCAdTdT-3′ [43]) and RPS27a (5′-CAGACAUUAUUGUGGCAAAdTdT-3′ or 5′-UUAGUCGCCUUCGUCGAGAdTdT-3′ [32]). Knockdown cells and cells treated with the GL2 control siRNA targeting firefly luciferase (5′-CGUACGCGGAAUACUUCGAdTdT-3′ [44]) were harvested 48 h after transfection. Cells were incubated for 18 h with low levels (5 ng/mL) of actinomycin D (ActD) to block ribosome biogenesis or 25 µM of MG132 (in ethanol) to inhibit the proteasome.

### 2.2. Western Blotting

Total cellular protein was separated by SDS polyacrylamide gel electrophoresis (SDS-PAGE) and transferred to a nitrocellulose membrane. Proteins were detected using ECL (Figure 1C, “FLAG”) or fluorescently labelled secondary antibodies and the LI-COR Odyssey system (all other Figures), and levels were determined using Image Quant software (G.E. Healthcare). Antibodies to detect the affinity tags on the fusion proteins were purchased from Sigma (anti-FLAG, F7425) or Berkeley Ab Company (anti-HA, MMS-101P), respectively. Other antibodies were: Anti-RPL7 (Abcam; ab72550), anti-p53 (Santa Cruz Biotechnology; sc-126), anti-karyopherin (loading control, Santa Cruz Biotechnology; sc11367), anti-RPL5 (Cell Signalling Technology; #14568). Anti-RPS19 antibodies were a kind gift from Phil Mason (Washington University School of Medicine, St. Louis, MO, USA).

### 2.3. Immunofluorescence

For immunofluorescence, U2OS T-REx Flp-In cells expressing the tagged protein of interest were plated on coverslips in a 24-well plate and treated with 1000 ng/mL tetracycline for 18 h. Cells were fixed in PBS containing 4% paraformaldehyde and then permeabilised in PBS/0.1% Triton. After blocking for 1–2 h using PBS/0.1% Triton/10% FCS solution, cells were incubated in the same solution containing the primary antibody for 1–2 h, followed by washes with PBS, then incubation with the secondary antibody for 1–2 h. After washing with PBS, and one wash with 0.01 ng/mL DAPI (4′,6-diamidino-2-phenylindole) diluted in PBS, the coverslips were mounted on a glass slide using Moviol. The cells were visualised using a Zeiss Axiovert 200M inverted microscope and analysed using the Axiovert software. For primary antibodies, a rabbit anti-FLAG antibody (Sigma Aldrich; F7425) and a mouse anti-HA antibody (Berkeley Ab Company; MMS-101P) were used. The secondary antibodies anti-Rabbit Alexa Fluor 555 (A-31572) and anti-Mouse Alexa Fluor 647 (A-31570) were both purchased from Invitrogen.

### 2.4. Gradient Analysis

For glycerol gradient analysis, U2OS T-REx Flp-In cells expressing the tagged protein of interest were treated with 1000 ng/mL tetracycline for 18 h. Whole-cell extracts prepared by sonication (approximately 8 × 10^6^ cells) were loaded on 10–40% glycerol gradients and separated by centrifugation (1.5 h at 52,000 rpm at 4 °C) using a swTi60 rotor (Beckman L7-80). The resultant fractions were analysed by SDS-PAGE and western blotting as described above.

### 2.5. RNA Analysis

RNA was extracted from cells using TRI reagent (Sigma-Aldrich) and separated on a 1.2% agarose-glyoxal gel and transferred to a nylon membrane by capillary blotting. DNA oligonucleotide probes specific to ITS1 (hybridising between site 2a and site 2, described previously [41]), RNase P (5′-CCTTCCCAAGGGACATGGGAGTGGAGTG-3′), the mature 18S rRNA (5′-GGGCGGTGGCTCGCCTCGCG-3′) and the mature 28S rRNA (5′-TGGTCCGTGTTTCAAGACGGGT-3′) were 5′-labelled using T4 polynucleotide kinase and ^32^P γATP. The probe used for ITS2 was generated by random-primed labelling from a PCR product (primers: forward 5′-GTGCGCGGCTGGGGGTTCCCTCGCAGG-3′ and reverse 5′-CCGGCACCCTTCCCCTTCCGGACC-3′) using ^32^P dATP as described previously [45]. All bands were detected using a PhosphorImager.

For RT-PCR, the RNA samples were treated with DNase Turbo before cDNAs were generated using oligo-dT primer and Superscript III (Invitrogen) reverse transcriptase. The specific cDNA fragments were then amplified using GoTaq polymerase (Promega) and the following primer pairs: GAPDH, 5′-GGTCGGAGTCAACGGATTTGGTCG-3′ and 5′-CGTTGTCATACCAGGAAATG-AGCTTGAC-3′; RPL40, 5′-AGGAGGGTATCCCACCTGACCAGC-3′ and 5′-CGAGCATAGCACTTGCGGCAGATC-3′; RPS27a, 5′-CCCTCGAGGTTGAACCCTCGG-3′ and 5′-GCCATTCTCATCCACCTTATAATATTTCAGG-3′. PCR products were separated by agarose gel electrophoresis using SYBR Safe stain (Invitrogen) and visualised using a PhosphorImager.

## 3. Results

### 3.1. Both the RPS27a and RPL40 Ubiquitin Fusion Precursors Are Efficiently and Rapidly Cleaved In Vivo

RPS27a and RPL40 are co-expressed as C-terminal fusions with ubiquitin and, as such, represent two of the four ubiquitin genes in mammals (Figure 1A; [18]). It is not yet completely clear when and how the fusion proteins are processed. It is possible that the separation of the ubiquitin and ribosomal protein moieties could take place during ribosome biogenesis. Since ubiquitin is important in ribosome biogenesis/function and excess ribosomal protein turnover, it may provide a means to co-regulate ribosomal protein production with ubiquitin levels in the cell [18]. To investigate this, we generated U2OS cells stably expressing either the RPS27a or RPL40 ubiquitin fusion proteins under the control of a tetracycline-regulated promoter. In each case, a FLAG-tag was added to the N-terminus of ubiquitin, and an HA-tag was added to the C-terminus of the ribosomal protein (Figure 1B).

These cells were first treated with a range of tetracycline concentrations (0–1000 ng/mL) to induce protein expression, and 18 h later, the cells were harvested, and expression of the ubiquitin–ribosomal protein fusions analysed by western blotting (Figure 1C). While commercially available antibodies were reported to detect RPS27a and RPL40 in prostate cancer cell lines [46], they did not function when tested in our hands. Therefore, levels of the endogenous ribosomal proteins could not be assessed. Cells expressing the RPS27a fusion protein produced a single, HA-tagged protein with an apparent molecular weight of ~18 kDa. We also observed a range of proteins conjugated to FLAG-tagged ubiquitin, with the most prominent band at ~25 kDa likely representing histone-ubiquitin. Importantly, the ~18 kDa band detected by the anti-HA antibody was not detected by the anti-FLAG antibody, indicating that this is the already cleaved RPS27a protein lacking the ubiquitin moiety. The HA-tagged RPS27a, which represents the cleaved ribosomal protein, appeared larger than predicted (9.4 kDa from the amino acid sequence). We assume that the highly basic nature of the sequence affects the migration of the protein through the polyacrylamide gel. Only background signals for both the anti-HA and anti-FLAG antibodies were detected in cells treated with tetracycline concentrations lower than 100 ng/mL. Upon treatment with 100 or 1000 ng/mL tetracycline, cells expressing RPL40 also produced a single, HA-tagged protein, albeit at lower levels compared to RPS27a, with an apparent molecular weight of about 12 kDa, and a similar distribution of FLAG-tagged, ubiquitinated proteins. Again, the HA-tagged RPL40, which is also a very basic protein, appeared larger than predicted from its amino acid sequence (6.2 kDa). No higher-molecular weight precursor proteins (~26.6 kDa for RPS27a or ~20.6 kDa for RPL40–considering the apparent MW of the two ribosomal proteins from the gel), detectable by both anti-HA and anti-FLAG antibodies were seen suggesting that processing of both ribosomal protein-fusion proteins is efficient.

Many ribosomal proteins are produced in excess, and the free proteins are quickly degraded by the proteasome [17]. The apparent lack of ubiquitin-fusion precursors upon tetracycline treatment could indicate rapid turnover of the excess non-cleaved precursor proteins (Figure 1C). However, it is also possible that the ubiquitin component might be cleaved before the proteasomal degradation of the excess ribosomal protein so that the ubiquitin can still be integrated into the ubiquitin pool. To test this, U2OS cells expressing tagged ubiquitin-RPS27a or RPL40, or the pcDNA5 empty vector, were treated for 18 h with 25 µM MG132, a proteasomal inhibitor [47]. A total of 1000 ng/mL tetracycline was added to the cells at the same time so that MG132 affected the expressed fusion proteins (Figure 1D).

Interestingly, the levels of HA-tagged RPS27a did not significantly change when the proteasome was blocked by treatment with MG132 (Figure 1E). In contrast, the levels of HA-tagged RPL40 were significantly increased after MG132 treatment. These results suggest that HA-RPL40, but not HA-RPS27a, is turned over by the proteasome. It should be noted that the level of overexpression may also affect the stability of the proteins. Due to the lack of functional antibodies, it was not possible to assess the ratios between HA-tagged and endogenous proteins in either case. However, previous studies using the tetracycline induction system for other ribosomal proteins, e.g., RPL5 and RPL11, found that proteins were expressed at a maximum of 20% of the endogenous protein levels [26]. We would therefore assume similar relative levels of expression for RPS27a and RPL40. Ubiquitin levels were not significantly affected by treatment with MG132 in either case or upon expression of the empty vector, indicating that ubiquitin is produced as normal. No obvious accumulation of either expected ubiquitin-fusion precursor was detected after inhibition of the proteasome by MG132 (Figure 1D), apart from a very faint ~25 kDa band seen for RPS27a, which may represent low levels of the fusion protein. These data suggest that the processing of the ubiquitin-fusion proteins occurs efficiently and, in the case of RPL40, before degradation of the excess cleaved protein.

### 3.2. The Function of RPL40, but Not RPS27a, Is Compromised by the Affinity Tag

The observed proteasomal degradation of HA-RPL40 suggests that the function of HA-RPL40 could be affected by the affinity tag used in this study, which might, in turn, affect its stability (Figure 1D,E).

To test if the affinity tag impacts on the cellular localisation of RPS27a or RPL40, immunofluorescence was performed (Figure 1F). U2OS cells containing either the pcDNA5 empty vector or constructs expressing the RPS27a or RPL40 fusion proteins were treated with 1000 ng/mL tetracycline for 18 h before staining. An anti-FLAG antibody was used to stain FLAG-tagged ubiquitin, and an anti-HA antibody was used to detect cleaved HA-tagged ribosomal proteins. U2OS cells containing the pcDNA5 empty vector showed a clear DAPI-staining marking on the nucleus but only a background signal when the anti-FLAG or the anti-HA antibodies were used (Figure 1F). In cells expressing the fusion proteins, FLAG-tagged ubiquitin was found mainly in the cytoplasm, with some traces in the nucleus but not in the nucleolus. This agrees with previous data showing that FLAG-tagged ubiquitin was found conjugated to both cytoplasmic and nuclear proteins (Figure 1C,D). HA-tagged RPS27a localised in both the cytoplasm and the nucleolus, while HA-tagged RPL40 was found mainly in the cytoplasm, together with a weak nucleoplasmic signal seen (Figure 1F). Overall, the localisation of the tagged proteins is consistent with what was previously seen for the endogenous RPS27a (https://www.proteinatlas.org/ENSG00000143947-RPS27A/subcellular; accessed on 11 April 2023) and RPL40 https://www.proteinatlas.org/ENSG00000221983-UBA52/subcellular, accessed on 11 April 2023) proteins in U2OS cells in the Human Protein Atlas resource project (https://www.proteinatlas.org/; accessed on 11 April 2023) [48].

Glycerol gradient analyses to separate free proteins from those associated with RNP complexes were performed to assess the integration of HA-tagged RPS27a and RPL40 into (pre-)ribosomal particles. For this, whole cell extracts from U2OS cells expressing HA-tagged RPS27a or RPL40 after the addition of tetracycline for 18 h were separated using a 10%–40% glycerol gradient and analysed by Western blotting (Figure 1G). HA-tagged ribosomal proteins were detected in the individual fractions using an anti-HA antibody, while an antibody against the endogenous ribosomal protein RPL7 was used as a marker for (pre-)60S complexes. HA-tagged RPS27a was mainly found in small ribosomal subunit (SSU) complexes (fractions 6–9), as expected, and no HA-tagged RPS27a was sedimented in the free protein fractions (fractions 1–5). While a small amount of HA-tagged RPL40 was detected in the expected large ribosomal subunit (LSU) complexes (fractions 10–15), the majority of the protein sedimented together with the SSU complexes (fractions 6–9) or in the free, non-ribosomal complex fractions (fractions 1–5).

The glycerol gradient, therefore, suggests that the affinity tag does not appear to affect the function of RPS27a. In contrast, the integration of HA-tagged RPL40 into (pre-)60S particles is impaired. Due to a lack of a functional antibody, we cannot determine whether this is due to the affinity tag on RPL40 or due to the protein being overexpressed, or both. Based on these observations, the cell line expressing HA-RPL40 was excluded from further analyses. However, the gradient data may provide an explanation for the observed instability of the HA-tagged RPL40 protein (Figure 1D,E). After cleavage from the fusion protein and without appropriate integration into (pre-)ribosomal complexes, HA-RPL40 is likely prone to degradation by the proteasome.

### 3.3. RPS27a Is Separated from Its Precursor in a Process Independent from Ribosome Biogenesis

**Figure 2:**
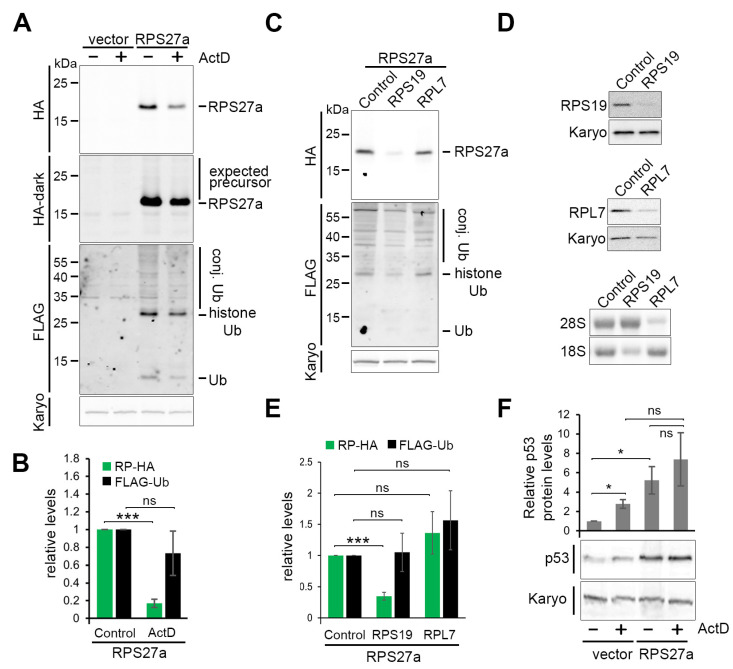
Processing of ubiquitin from the Ub-RPS27a precursor occurs independently of ribosome biogenesis. (**A**) U2OS cells stably expressing the RPS27a ubiquitin fusion precursor protein or the empty pcDNA5 vector, were incubated for 18 h with 1000 ng/mL tetracycline in the absence (-) or presence (+) of 5 ng/mL ActD. Proteins from these cells were analysed by western blotting using antibodies that recognise the HA-tag, the FLAG-tag or karyopherin (Karyo; loading control), as indicated on the left of each panel. HA-dark is a stronger exposure of the HA-signal to facilitate visualisation of the expected precursor. The positions of the various proteins, and expected ubiquitin-ribosomal protein fusions, are indicated on the right of the panels. (**B**) The ribosomal protein-HA (RP, green bars) and FLAG-Ub (black bars) western blot signals from (**A**) were quantitated and normalised to karyopherin and plotted. (**C**) The RPS27a ubiquitin fusion precursor protein was expressed with 1000 ng/mL tetracycline in U2OS cells transfected with the control siRNA or siRNAs targeting either RPS19 or RPL7 (indicated above each lane). Proteins from these cells were harvested after 48 h and analysed by western blotting as described in (**A**). (**D**) U2OS cells stably expressing the RPS27a ubiquitin fusion precursor protein, as described in (**C**), were transfected with siRNAs to deplete either RPS19 or RPL7. Total protein was analysed by western blotting using antibodies specific to the ribosomal proteins or karyopherin (Karyo; loading control), as indicated on the left of each panel. The impact of the siRNAs on mature rRNA levels was assessed by glyoxal/agarose gel electrophoresis and northern blotting using probes specific to the mature 18S and 28S rRNAs (as indicated on the left). (**E**) The ribosomal protein-HA (RP, green bars) and FLAG-Ub (black bars) western blot signals from (**C**) were quantitated and normalised to karyopherin and plotted. (**F**) Proteins from (**A**) were analysed by western blotting using antibodies that recognise p53 or karyopherin (Karyo; loading control), as indicated on the left. Western blot signals were quantitated, normalised to karyopherin and plotted. For all graphs, quantification is based on 3 independent repeats. Error bars indicate SEM. * < 0.05, *** < 0.001, ns > 0.05 (not statistically significant).

To test whether ribosome biogenesis is important for the cleavage of the ubiquitin-RPS27a fusion protein, we treated cells expressing affinity-tagged RPS27a, alongside cells containing the empty pcDNA5 vector, with 1000 ng/mL tetracycline for 18 h to induce protein expression. Low levels (5 ng/mL) of Actinomycin D (ActD) were added to block rRNA transcription by RNA polymerase I and thus abolish ribosome production. ActD and tetracycline were added at the same time so that all the tagged proteins were produced after ribosome production had been blocked. In this situation, newly synthesised ribosomal proteins for both subunits are unstable [17]. Treatment of cells with ActD resulted in a five-fold decrease in HA-tagged RPS27a but no significant change in FLAG-ubiquitin levels (Figure 2A,B). Again, no ubiquitin–ribosomal protein fusion precursor band was observed.

To further analyse the role of ribosome biogenesis in the processing of the ubiquitin-RPS27a fusion protein, we tested the effect of depleting either a small (RPS19) or large (RPL7) ribosomal subunit protein on the accumulation of HA-RPS27a and FLAG-ubiquitin. Cells were simultaneously transfected with the respective siRNA to mediate protein knockdown and treated with 1000 ng/mL tetracycline to induce expression of the fusion protein for 48 h (Figure 2C,E). The knockdown efficiency of RPS19 and RPL7 was monitored by Western blotting. Northern blotting was performed to demonstrate that the knockdown of each ribosomal protein causes a strong decrease in the levels of the mature 18S or 28S rRNAs, respectively (Figure 2D).

Knockdown of RPS19 resulted in an about three-fold decrease in HA-tagged RPS27a levels, while knockdown of RPL7 had no impact (Figure 2C,E). Neither knockdown resulted in the accumulation of the ubiquitin-RPS27a precursor or impacted FLAG-ubiquitin production. Our data, therefore, show that HA-RPS27a is dependent on SSU, but not LSU production, while the production of ubiquitin appears independent of ribosome biogenesis. These observations suggest that cleavage of the ubiquitin-RPS27a precursor does not require ribosome biogenesis and that ubiquitin and HA-RPS27a accumulate independently of one another.

### 3.4. Cells Expressing the Affinity-Tagged Ubiquitin-RPS27a Precursor Exhibit Higher p53 Levels, Which Do Not Change in Response to ActD Treatment

Overexpression of RPS27a and its ubiquitin-fusion precursor has been shown to inhibit MDM2-mediated p53 degradation in U2OS cells [32]. To test whether this is also the case in our in vivo system, we treated cells expressing HA-tagged RPS27a, alongside those containing the empty pcDNA5 vector, with 1000 ng/mL tetracycline for 18 h to induce protein expression. Low levels (5 ng/mL) of Actinomycin D (ActD) were added at the same time, which was previously demonstrated to cause p53 stabilisation through blocking ribosome biogenesis [26].

Over-expression of RPS27a resulted in a significant five-fold p53 increase compared to the cells containing the empty pcDNA5 vector (Figure 2F). Treatment with ActD in U2OS cells containing the empty vector resulted in a slightly lower but also significant two-fold p53 increase compared to the non-treated cells. Interestingly, ActD treatment in U2OS cells expressing HA-tagged RPS27a did not result in a significant change in p53 levels as compared to the non-ActD-treated U2OS cells expressing the HA-tagged ribosomal protein. Likewise, p53 levels did not significantly differ between ActD-treated cells expressing HA-tagged RPS27a or the empty vector. These results confirm that the expression of HA-tagged RPS27a results in p53 stabilisation, which is consistent with the previous study [32]. However, the data also indicate that over-expression of RPS27a does not further enhance p53 stabilisation seen after ActD-induced ribosome biogenesis defects.

### 3.5. Knockdown of RPS27a or RPL40 Leads to p53 Stabilisation in MCF7 and LNCaP Cells, but Not in U2OS Cells

Having established the effect of overexpressing RPS27a on p53 levels, we next investigated the impact of reducing the levels of RPS27a or RPL40 on p53 signalling in U2OS cells. Knockdown of many ribosomal proteins has been shown to induce p53 through defects in ribosome biogenesis and the 5S RNP [28,29,37]. Indeed, knockdown of either RPS27a or RPL40 caused significant p53 stabilisation in HCT116 cells [29] and A549 cells [28,39]. However, in the aforementioned study describing the effect of RPS27a overexpression in U2OS cells, RPS27a knockdown did not induce p53 but instead blocked p53 stabilisation in response to inhibiting ribosome biogenesis using either ActD or 5FU [32].

**Figure 3:**
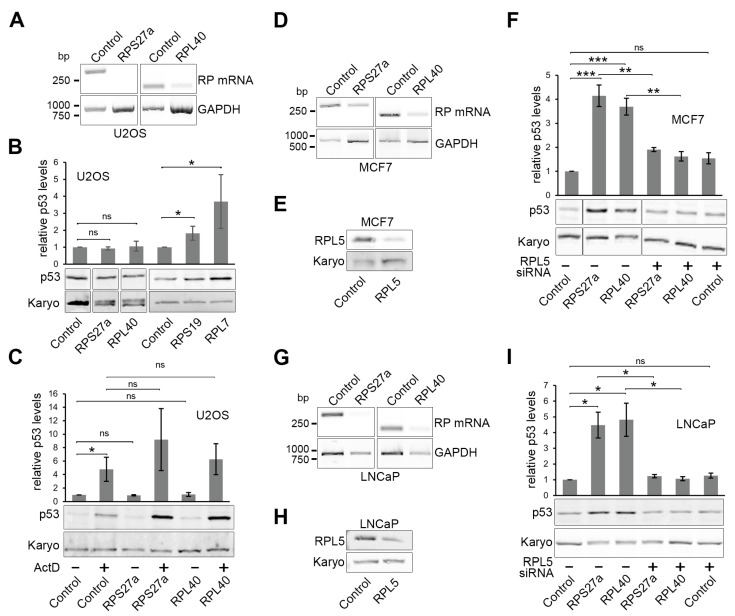
Knockdown of RPS27a and RPL40 results in p53 stabilisation in MCF7 and LNCaP cells, but not in U2OS cells. (**A**,**D**,**G**) RNA was extracted from U2OS (**A**), MCF7 (**D**) or LNCaP (**G**) cells depleted of RPS27a or RPL40 by RNAi or control cells (indicated at the top of each lane), harvested 48 h post-transfection, and analysed by RT-PCR using primers specific for the RPS27a, RPL40 or GAPDH (loading control) mRNAs (indicated to the right of each panel). PCR products were separated by agarose gel electrophoresis and SYBR Safe-stained bands detected using a PhosphorImager. The positions of the DNA ladder markers are shown on the left of the panels. (**B**) RPS27a, RPL40, RPS19 or RPL7 were knocked down in U2OS cells and harvested after 48 h. Proteins isolated from these cells and control cells (indicated below each lane) were analysed by western blotting using antibodies that recognise p53 and karyopherin (Karyo; loading control). The p53 levels, relative to the control, were calculated for each lane and plotted. (**C**) RPS27a and RPL40 were knocked down in U2OS cells for 48 h and either untreated (−) or treated (+) with 5 ng/mL ActD for 18 h (added 30 h post-transfection). Proteins isolated from these cells and control cells (indicated below each lane) were analysed by western blotting using antibodies that recognise p53 and karyopherin (Karyo; loading control). The p53 levels, relative to the control -ActD lane, were calculated for each lane and plotted. (**E**,**H**) MCF7 (**E**) or LNCaP (**H**) cells were transfected with the control siRNA or a siRNA targeting RPL5, and proteins were isolated after 48 h and analysed by western blotting using antibodies that recognise RPL5 or karyopherin (Karyo; loading control). (**F**,**I**) RPS27a or RPL40 were knocked down in MCF7 (**F**) or LNCaP (**I**) cells for 48 h, either alone or together with RPL5, and the levels of p53 determined by western blotting as described in (**B**). The p53 levels, relative to those seen with control cells, were calculated for each lane and plotted. The siRNAs used are indicated at the bottom of the lanes and the protein detected by the antibody on the left of each panel. For all graphs, quantification is based on 3 independent repeats. Error bars indicate SEM. * < 0.05, ** < 0.01, *** < 0.001, ns > 0.05 (not statistically significant).

To shed light on these conflicting reports and to clarify whether knockdown of RPS27a and/or RPL40 inhibits or causes p53 accumulation, we transfected U2OS cells with siRNAs targeting RPS27a or RPL40 or with control siRNAs targeting firefly luciferase and analysed p53 levels by Western blotting (Figure 3). Since no functional commercial antibodies were available for RPS27a and RPL40, the efficiency of the knockdown was confirmed by RT-PCR (Figure 3A). In each case, a reduction in RT-PCR signal, and therefore ribosomal protein mRNA levels, relative to the levels of the GAPDH mRNA, was observed for the cells transfected with the siRNAs targeting the ribosomal protein mRNA relative to cells transfected with the control siRNA.

Confirming the earlier observation [32], the knockdown of RPS27a did not result in p53 accumulation in U2OS cells (Figure 3B). The same result was observed for RPL40. Notably, the knockdown of RPS19 or RPL7 caused a significant ~two-fold or ~four-fold increase in p53 levels, respectively, which is consistent with previous studies in HCT116 cells [29] and A549 cells [28].

The fact that the knockdown of RPS27a and RPL40 does not induce p53 in U2OS cells suggests that, as previously reported for RPS27a [32], they may be involved in p53 signalling. If this is the case, their knockdown should block p53 stabilisation upon ActD treatment. To test whether RPS27a or RPL40 is important for p53 stabilisation, U2OS cells were transfected with either the RP-targeting siRNAs or the control siRNA for 48 h. Cells were also treated with low levels (5 ng/mL) of ActD (for 18 h, added 30 h post-transfection) to induce p53, and p53 levels were determined by western blotting (Figure 3C). ActD treatment resulted in a significant, four-fold to five-fold increase in p53 levels. Surprisingly, the knockdown of neither RPS27a nor RPL40 had any impact on p53 stabilisation by ActD. Therefore, our data show that RPS27a and RPL40 are not needed for p53 stabilisation in U2OS cells upon ActD-induced defects in ribosome biogenesis, which is different from the previous report [32].

Next, we wanted to further investigate whether the impact of RPS27a or RPL40 knockdown on p53 signalling could indeed be cell-type dependent. For this, we chose MCF7 (breast cancer) and LNCaP (prostate cancer) cancer cell lines, both of which have wild-type p53 and are routinely used to study p53-dependent cellular signalling. Knockdown efficiency for RPS27a and RPL40 in MCF7 and LNCaP cells was determined by RT-PCR (Figure 3D,G), while the impact on p53 signalling was analysed by western blotting (Figure 3F,I).

In both MCF7 and LNCaP cells, knockdown of either ribosomal protein induced a robust, three-fold to five-fold p53 stabilisation (Figure 3F,I). This demonstrates that as in HCT116 [29] and A549 [28,39] cells, the knockdown of RPS27a or RPL40 stabilises p53 in both MCF7 and LNCaP cells. Knockdown of ribosomal proteins generally induces p53 through the 5S RNP and p53 stabilisation is blocked by the co-depletion of either of the 5S RNP proteins, RPL5 or RPL11 [26,27]. To determine whether this is also the case with RPS27a and RPL40, we co-depleted the 5S RNP protein RPL5 (Figure 3F,I) with either RPS27a or RPL40 in both MCF7 and LNCaP cells. Treatment of the cells with siRNAs depleting RPL5 resulted in a reduction of RPL5 levels to 40–50% of those treated with control siRNAs (Figure 3E,H). Importantly, co-depletion of RPL5 significantly decreased p53 stabilisation caused by the knockdown of either RPS27a or RPL40 (Figure 3F,I). Note that in both MCF7 and LNCaP cells, the knockdown of RPL5 alone did not significantly increase p53 levels as expected since RPL5 is essential for p53 signalling. We also did not observe a decrease in p53 levels with knockdown of just RPL5, as we had earlier published in U2OS cells [26], which may reflect that RPL5 is less important for maintaining p53 levels in MCF7 and LNCaP cells under non-stress conditions.

Taken together, our data demonstrate that the knockdown of RPS27a or RPL40 stabilises p53 via the 5S RNP-MDM2 pathway in both MCF7 and LNCaP cells but not in U2OS cells. We further conclude that neither RPS27a nor RPL40 are needed for p53 signalling in response to ribosome biogenesis defects in either cell line.

### 3.6. RPS27a and RPL40 Are Important for Ribosome Production in U2OS, MCF7 and LNCaP Cells

**Figure 4:**
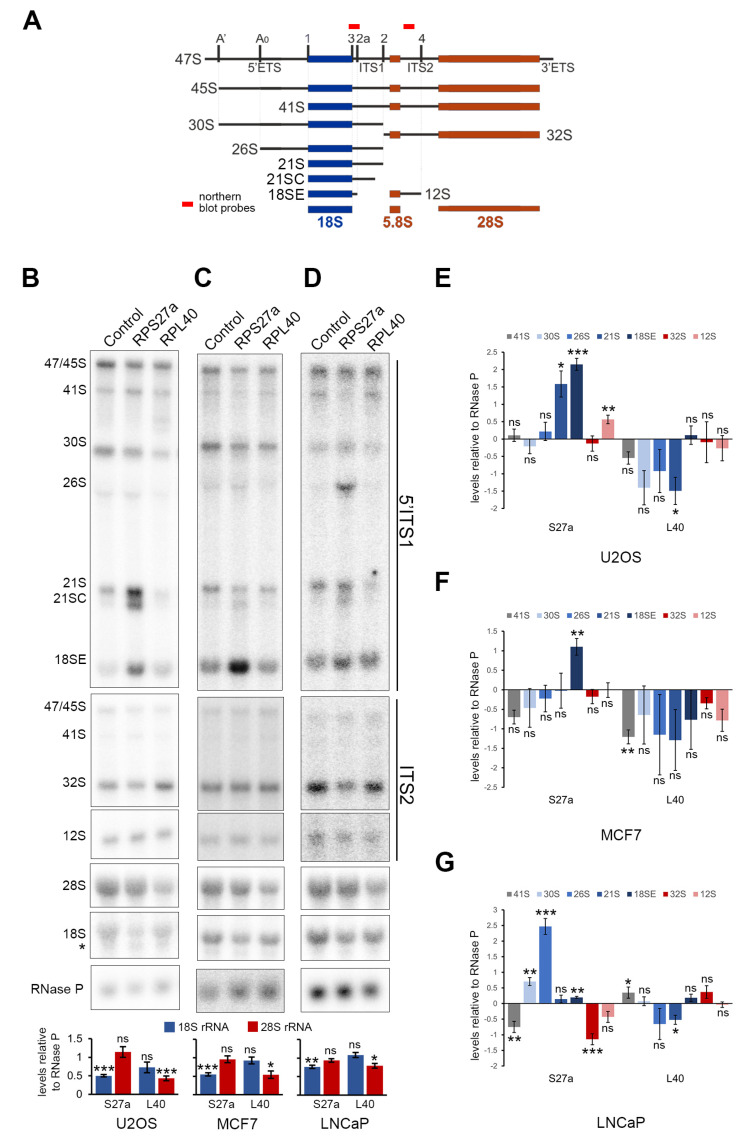
RPS27a and RPL40 are needed for pre-rRNA processing in U2OS, MCF7 and LNCaP cells. (**A**) Schematic representation of the pre-rRNA processing intermediates with the external transcribed spacers (ETS) and internal transcribed spacers (ITS) and processing/cleavage sites indicated. The relative positions of the northern blot probes are indicated above the 47S pre-rRNA. (**B**–**D**) RNA was extracted from control U2OS (**B**), MCF7 (**C**) and LNCaP (**D**) cells or cells depleted by RNAi of RPS27a or RPL40 for 48 h, separated by glyoxal/agarose gel electrophoresis and then analysed by northern blotting using probes specific to the 5′ end of ITS1 (5′ITS1), ITS2, the mature 18S and 28S rRNAs, or RNase P (loading control). The identities of the RNAs and pre-rRNAs are indicated on the left. * indicates a putative 18S degradation intermediate in U2OS cells (**B**). The levels of the mature 18S and 28S rRNAs after knockdown of RPS27a and RPL40, respectively, were determined and plotted relative to RNase P levels in the control cells. (**E**–**G**) The levels of SSU and LSU precursors after knockdown of RPS27a and RPL40 in U2OS (**B**), MCF7 (**C**) and LNCaP (**D**) cells, respectively, were determined. Bar charts show log2 ratios of precursors relative to RNase P levels in the control cells. For all graphs, quantification is based on 3 independent repeats. Error bars indicate SEM. * < 0.05, ** < 0.01, *** < 0.001, ns > 0.05 (not statistically significant).

We next asked if the knockdown of RPS27a and RPL40 may affect different stages of pre-rRNA processing in U2OS, MCF7 and LNCaP cells (Figure 4) and whether this may influence whether p53 is stabilised upon protein knockdown or not. This analysis is important since the knockdown of RPS27a in HeLa and HCT116 cells has already shown somewhat different effects on pre-rRNA processing. RPS27a was shown to be important for intermediate stages of 18S rRNA maturation in HeLa cells (mainly 26S and 21S accumulation upon knockdown) [49] but needed for intermediate and earlier stages (21S/21SC and 41S pre-rRNA accumulation upon knockdown) in HCT116 cells [29].

U2OS, MCF7 and LNCaP cells were transfected with siRNAs designed to knockdown RPS27a and RPL40, RNA was extracted and the effect on mature 18S and 28S rRNA levels and pre-rRNA processing was analysed by glyoxal agarose gel electrophoresis followed by northern blotting.

Northern blot analysis revealed that knockdown of RPS27a resulted in a significant drop in mature 18S rRNA levels, relative to RNase P RNA, compared to control knockdown cells for each cell line, with no significant change in 28S rRNA levels (Figure 4B–D). Interestingly, the reduction in 18S levels also coincided with an increase in the detection of a shorter product, presumably an 18S degradation intermediate, in U2OS cells (Figure 4B, mature 18S probe). Knockdown of RPL40 resulted in a major decrease in mature 28S rRNA levels (Figure 4B–D), with mature 28S rRNA levels dropping to about 50% of those seen in control cells in U2OS and MCF7 cells (Figure 4B,C). In LNCaP cells, the impact on 28S rRNA levels upon RPL40 knockdown was less severe, although statistically significant (Figure 4D).

Notably, while depletion of RPS27a always resulted in a defect in the mid to late stages of 18S rRNA processing, the change in pre-rRNA intermediate levels were different in each cell line tested (Figure 4B–D). In U2OS cells, there were no changes in 41S, 30S or 26S pre-rRNA levels, but significant increases in 21S and 18SE pre-rRNA levels, accompanied by the appearance of the aberrant 21SC pre-rRNA (Figure 4B, 5′ITS1 probe and Figure 4E). In MCF7 cells, on the other hand, depletion of RPS27a resulted in an apparent but not significant reduction in 41S levels and no significant changes in the 30S, 26S or 21S pre-rRNAs. However, the appearance of 21SC and, most strikingly, a significant increase in the levels of the 18SE pre-rRNA were observed (Figure 4C, 5′ITS1 probe and Figure 4F). In LNCaP cells, knockdown of RPS27a resulted in a significant reduction in 41S pre-rRNA levels, significant accumulation of the 30S, 26S and 18SE precursors and the appearance of 21SC, while 21S levels were unchanged (Figure 4D, 5′ITS1 probe and Figure 4G). Surprisingly, the knockdown of RPS27a in U2OS cells also resulted in a significant accumulation of the 12S precursor, while RPS27a knockdown in LNCaP cells caused a significant reduction in 32S levels and an apparent reduction in the levels of the 12S pre-rRNA that are both linked to LSU production. However, these defects did not appear to be strong enough to result in significant changes in the levels of the mature 28S rRNA, and they were not seen in MCF7 cells (Figure 4B–D, ITS2 probe and Figure 4E–G). Contrary to the strong impact of the RPL40 knockdown on mature 28S rRNA levels, Northern blotting revealed no significant change in the levels of the LSU pre-rRNAs in either cell line (Figure 4B–D, ITS2 probe and Figure 4E–G). However, RPL40 knockdown surprisingly led to a significant reduction of the 21S SSU precursor in U2OS and LNCaP cells. In contrast, levels of the 41S pre-rRNA were significantly decreased in MCF7 cells and increased in LNCaP cells (Figure 4B–D, 5′ITS1 probe and Figure 4E–G).

Taken together, our data indicate that RPS27a and RPL40 are essential for the production of their respective (18S or 28S) mature rRNA in all cell lines tested. However, the accumulation of specific SSU and LSU precursors seen upon knockdown varies between different cell types.

## 4. Discussion

The ribosomal proteins RPS27a and RPL40 are produced as fusion proteins with an N-terminal ubiquitin in all eukaryotes, and their genes represent two of the four ubiquitin genes in the mammalian genome [18]. To investigate their production and function in human cells, we generated U2OS cells stably expressing either the RPS27a or RPL40 ubiquitin fusion proteins. In each case, a FLAG-tag was added to the N-terminus of ubiquitin, and an HA-tag added to the C-terminus of the ribosomal protein. While the affinity-tag on either protein did not appear to interfere with their cellular localisation, HA-tagged RPL40 was unstable and showed impaired integration into (pre-)ribosomal complexes. The cell line expressing the RPL40 fusion protein was therefore excluded from further analysis. A previous study on RPL40 in hepatoma cells utilised a C-terminal RFP tag on RPL40 in their fusion construct, which also did not affect the cellular localisation of the cleaved RPL40 protein [25]. However, the integration of RPL40-RFP into (pre-)ribosomal complexes was not assessed. Notably, adding an HA-tag to the N-terminus of yeast Rpl40 did not interfere with its assembly into ribosomes [50], suggesting that an N-terminal HA tag on RPL40 may be more suitable for future analysis in human cells.

To our surprise, we never saw the accumulation of the ubiquitin–ribosomal fusion proteins, not even in the case of RPS27a, when ribosome biogenesis was blocked. Blocking ribosome biogenesis also only affected the production of the ribosomal protein but not ubiquitin. This strongly suggests that the processing of the RPS27a fusion protein, and presumably also the RPL40 fusion protein, is independent of ribosome production, as has been proposed in yeast [19,51,52]. Indeed, the apparently rapid nature of this processing event suggests that cleavage takes place co-translationally on the ribosome. The identification of the enzyme/deubiquitinase responsible is needed before this processing event can be studied in more detail. While we have shed new light on the production and function of the two ribosomal proteins in ribosome production and cellular signalling, it remains unclear why these proteins are produced, throughout eukaryotes, as ubiquitin fusion proteins that require post-translational processing.

We also demonstrate that RPS27 and RPL40 are essential for the production of their respective subunits in all cell lines tested. Surprisingly, and consistent with a previous study on RPS27a in U2OS cells [32], the knockdown of either protein did not cause p53 stabilisation in U2OS cells. In addition, our experiments revealed that neither RPS27a nor RPL40 is needed for p53 stabilisation in U2OS cells upon ActD-induced defects in ribosome biogenesis, which is different from the previous report on RPS27a [32]. However, and again consistent with the previous study [32], p53 stabilisation was observed upon expression of the RPS27a fusion protein. Taken together, the combined data suggest that RPS27a may play a role in p53 signalling, at least in U2OS cells, beyond its being an essential component of the ribosome. Further experiments involving the use of other cell lines (see below) are essential to clarify its role in cell signalling and how this is linked to ribosome biogenesis.

Contrary to what was seen in U2OS cells, knockdown of RPS27a and RPL40 led to p53 stabilisation in MCF7 and LNCaP cells, consistent with previous studies in HCT116 cells and A549 cells and what was seen for the majority of other ribosomal proteins. [28,29,39] We cannot explain why the knockdown of either RPS27a or RPL40 does not induce p53 in U2OS cells. This could be due to cell-line-specific differences in the expression levels of RPS27a and RPL40, but a comparison of the available expression data from cell lines collated in the Human Protein Atlas resource project (https://www.proteinatlas.org/; accessed on 11 April 2023) [48] does not support this hypothesis.

Interestingly, our data on pre-rRNA processing defects seen upon RP27a and RPL40 knockdown is somewhat similar to what was observed in yeast. Depletion of Rps31 (yeast RPS27a) showed some defects in the mid to early steps of yeast 18S maturation but blocked the final processing of the cytoplasmic 20S precursor [51,53] (equivalent of human 18SE). Likewise, yeast Rpl40 depletion resulted in only a minor depletion of the mature LSU rRNAs [54]. However, in yeast, an increase in the initial transcript, 35S (47S in humans), and a slight delay in ITS2 processing [50] was seen upon Rpl40 depletion.

It is quite striking that while both RPS27a and RPL40 knockdowns had an impact on the levels of their respective mature rRNA in all cell lines tested, only RPS27a knockdown caused a strong accumulation of mostly mid to late SSU pre-rRNAs. In contrast, RPL40 depletion had no significant impact on mid to late LSU precursor RNA levels. Again, our data is somewhat in agreement with the data in yeast which showed minimal impact on pre-rRNA levels upon Rpl40 depletion [50,54]. It has also been shown that Rpl40 assembles with pre-60S complexes late in the cytoplasm, which is likely similar in human cells after most pre-rRNA cleavages have already occurred [50]. Moreover, earlier work in HCT116 cells had revealed that the knockdown of a few other LSU ribosomal proteins (e.g., RPL27a (uL15) and RPL26 (uL24)) also significantly impacted mature 28S rRNA levels with minimal change in pre-rRNA levels [29]. In these instances, and likely similar to what we observed upon RPL40 knockdown in three different cell lines, we believe that the ribosomal proteins are more important for the stability of the mature LSU than for any of the individual pre-rRNA processing steps.

Finally, we observed differences in the pre-rRNA processing defects seen upon knockdown of RPS27a and RPL40 between U2OS, MCF7 and LNCaP cells and also compared to those previously published in HeLa and HCT116 cells [29,49]. Firstly, the significant SSU or LSU processing defects seen with respect to their own subunit varied between different cell lines. RPS27a knockdown surprisingly also caused significant defects in LSU maturation and changes in the 41S pre-rRNA levels, while both 41S and 21S SSU precursor levels were significantly altered upon RPL40 depletion, and this was again different in different cells. These could reflect cell-line-specific differences in the functions of these proteins in ribosome biogenesis. This is likely due to the numerous and distinct mutations in genes encoding both ribosomal proteins and ribosome biogenesis factors, which may directly impact pre-rRNA processing [55,56]. However, analysis of the mutations in the available exome sequencing databases revealed no obvious explanation for the cell-line specific data we observed, both with respect to p53 signalling and ribosome biogenesis. We and others have previously reported that mammalian pre-rRNA processing pathways vary slightly between different cell lines or tissues, for example, with respect to 5′ETS and ITS1 processing [57,58]. However, we have not, with the exception of the RPS27a and RPL40 knockdowns described here, observed significant cell-line specific differences in the pre-rRNA processing defects seen with other ribosomal protein knockdowns (our unpublished data). Further characterisation of the genetic background of each cell line will help to understand the impact of individual ribosomal protein knockdowns on both ribosome biogenesis and p53-dependent cellular signalling.

## Data Availability

Not applicable.
